# Antiproliferative Effects of Polar Extracts of the Aerial Parts of *Fuchsia standishii* J. Harrison

**DOI:** 10.3390/plants14243779

**Published:** 2025-12-11

**Authors:** María I. Ramírez, Aday González-Bakker, Adam N. Khan, Adrián Puerta, José M. Padrón

**Affiliations:** 1 BioLab, Instituto Universitario de Bio-Orgánica Antonio González (IUBO-AG), Universidad de La Laguna, P.O. Box 456, E-38200 La Laguna, Spain; miramirez@utpl.edu.ec (M.I.R.); agonzaba@ull.es (A.G.-B.); alu0101224077@ull.edu.es (A.N.K.); apuertaa@ull.es (A.P.); 2 Departamento de Ciencias de la Salud, Universidad Técnica Particular de Loja, San Cayetano Alto, Loja 1101608, Ecuador

**Keywords:** *Fuchsia standishii* J.Harrison, ethnopharmacology, anti-cancer, apoptosis, genotoxicity, UHPLC-DAD-MS^3^

## Abstract

*Fuchsia standishii* J.Harrison is a species widely used in traditional medicine in southern Ecuador for treating various ailments, including high blood pressure, as an antacid and a relaxant. The pharmacological basis for these traditional uses is unknown. Given the reported anti-inflammatory and cytotoxic properties of the *Onagraceae* family, we investigated the plant’s potential for addressing chronic conditions. This study explored the bioactive potential of polar extracts from the aerial parts of *F. standishii*, focusing on antiproliferative activity against a panel of human tumor cell lines (A549, HBL-100, HeLa, SW1573, T-47D). The plant material was sequentially extracted and partitioned into nine fractions. All fractions were screened for antiproliferative activity, and the most active fractions were further evaluated for their mechanism of cell death (apoptosis/necrosis), genotoxicity, and induction of oxidative stress. Specialized metabolites in the fractions were characterized using UHPLC-DAD-MS^3^ analysis. *F. standishii* extracts showed potent antiproliferative activity. The dichloromethane fraction (MW_D_) was the most active (GI_50_ range: 8.5–39 µg/mL), demonstrating the ability to induce apoptosis in tumor cells and cause genotoxic damage linked to oxidative stress. The UHPLC-DAD-MS^3^ analysis successfully characterized the specialized metabolites present in the active fractions. The initial aqueous extract yielded a total of 47 secondary metabolites, 15 of which remained unassigned. *F. standishii* possesses a promising pharmacological profile that extends beyond its documented traditional uses. The MW_D_ fraction represents a plausible source of novel anti-cancer agents due to its ability to induce apoptosis, supporting further bioguided investigation of this ethnobotanically relevant species.

## 1. Introduction

The use of natural remedies and traditional medicines to manage diseases is extensive [1]. In this context, Ecuador stands out. It is a country rich in biodiversity, and a large part of its population lacks access to the public health system. For these communities, nature and ancestral practices remain the almost exclusive source for disease treatment [1,2]. Over 25,000 plant species exist in Ecuador, with the Andean region hosting the majority [3]. The province of Loja in southern Ecuador is a biologically complex area. Here, the Andes Mountain range has its lowest altitudinal distribution, known as the Huancabamba Deflection. This contributes to a highly particular physiography that includes dry valleys, cloud forests, and Amazonian moors and forests, differing significantly from those in the north of the country [4]. Ecuador’s rich biodiversity attracted the interest of prominent naturalists like La Condamine (1738), Humboldt (1802), and Litle (1978). Their observations described Loja as the “botanical garden of Ecuador.” An example of the significance of the southern region’s biodiversity is the presence of *Cinchona officinalis*. This plant grows in the mountains of Loja, and its use to treat malaria dates back to pre-Hispanic occupation; *C. officinalis* continues to be used worldwide [4,5].

**Figure 1:**
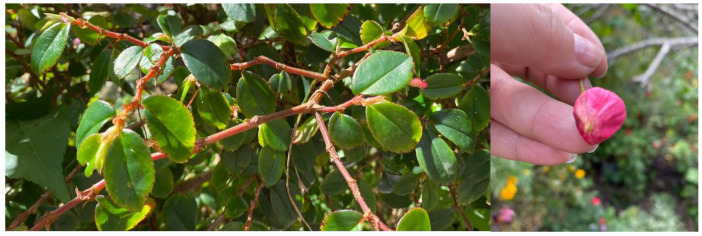
Aerial parts of *Fucshia standishii* (pena pena) used in traditional medicine in southern Ecuador.

Considering the persistent interest in obtaining new medicinal products and the vast biodiversity of Ecuador (particularly in Loja), the present study focuses on *Fuchsia standishii* J.Harrison (syn. *F. hybrida*, Figure 1), one of the most popular and used species in the province, commonly known as “pena pena” or queen’s earrings. *F. standishii* is distributed across Mexico, Central, and South America, originating from the hybridization of three South American *Fuchsia* species: *F. fulgens* Moc. et Ses., *F. magellanica* Lam., and *F. corymbiflora* [6].

While the traditional uses of *F. standishii* are primarily related to symptomatic relief (e.g., blood pressure, antacid, relaxant), the presence of flavonoids, tannins, and triterpenoids in the *Onagraceae* family suggests a wide range of potential pharmacological activities, including anti-inflammatory and cytotoxic effects [7]. Given that chronic inflammation is a known precursor to many chronic diseases, including certain cancers, we hypothesize that the traditionally used extracts may possess antiproliferative properties. Specifically, *F. magellanica*, a parent species of *F. standishii*, is prominent in traditional medicine for treating boils, skin inflammation, and for its diuretic and antipyretic effects [8]. It is notably rich in anthocyanins [9]. In vitro studies have demonstrated its pharmacological potential, showing cytotoxic effects on fibroblast (3T3) and keratinocyte (HaCaT) cell lines [10], and significant inhibition of cell proliferation in human breast cancer cell cultures (MCF7 and MDA-MB-231) [11].

Previous studies on the genus *Fuchsia* have established a framework of bioactivity and phytochemistry. *F. magellanica* stands out, as its extracts have demonstrated a potent intestinal and uterine antispasmodic effect, acting as a non-competitive inhibitor of contraction, with IC_50_ values up to 152.9 ± 29.1 μg/mL in the inhibition of Ca^2+^ influx. This bioactivity is related to the presence of flavonoids (quercetin and kaempferol) and a complex composition of anthocyanins. The flowers of *F. magellanica* and *F. fulgens* var. Variegata have been reported to contain up to thirteen types of anthocyanidins: 3,5-diglucosides, 3-monoglucosides, and 3-(2″-galloylglucosides), including peonidin 3-O-(2″-O-galloyl-β-glucopyranoside). Purple hues correlate with a high content of malvidin 3,5-diglucoside. However, despite this rich characterization, the biological activity of the *F. standishii* J. Harrison and its non-floral aerial parts has not been reported.

Since similar biological activity studies are limited in the literature for *F. standishii*, we were encouraged to explore its bioactive potential beyond the reported ethnobotanical applications. Therefore, the aim of this study was to explore the bioactive potential of the polar extracts from the aerial parts of *F. standishii* (excluding the flowers) against human solid tumor cell lines, as this represents a robust, preliminary screening method for identifying novel therapeutic agents [12,13], particularly given the precedence of cytotoxic activity in the related species, *F. magellanica*. Our work seeks to determine whether this hybrid possesses significant biological potential (antiproliferative, ROS, and DNA damage) that would validate its ethnomedicinal use.

## 2. Results

### 2.1. Preparation of Extracts and Their Fractionation

The extraction process of the dried aerial parts of *F. standishii* (100 g) was performed sequentially using, in order, water, methanol, and dichloromethane. These solvents allow to obtain a vast range of specialized metabolites (range of polarities and bioactivities), which could be difficult to obtain using a single extraction [14]. The three mother liquors underwent evaporation of the volatile solvents (methanol and dichloromethane) followed by resuspension in water (up to 300 mL) of the remains. The next step involved a liquid–liquid extraction. For this process, two solvents were considered. On the one hand, dichloromethane to obtain low polarity substances. On the other, ethyl acetate to access substances of medium and high polarity. Thus, the three water phases (derived from the mother liquors) were extracted sequentially (1:1, *v*/*v*) with dichloromethane and ethyl acetate. In total, from each mother liquor, three liquid phases were obtained. Namely, dichloromethane (D), ethyl acetate (E) and water (W). Next, the volatile solvents were removed by evaporation and water was removed by lyophilization. For each sample, the dry mass was weighed. Table 1 shows the calculated extraction yield expressed in percentage. In this study, the polar extracts from the aerial parts of *F. standishii* gave the highest yields, indicating the prevalence of polar compounds in the plant (MW_W_ yield = 26.8%).

### 2.2. Antiproliferative Activity

**Figure 2:**
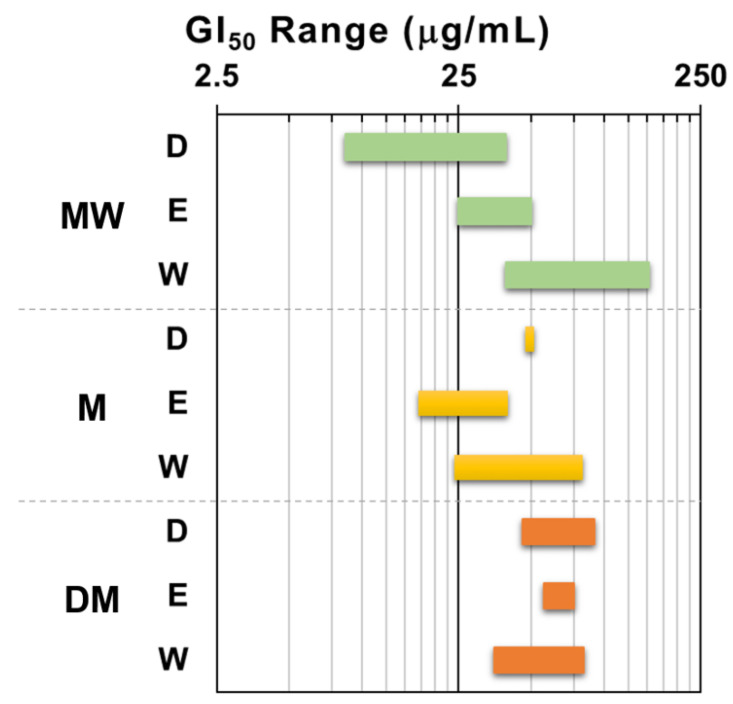
Range plot of antiproliferative activity (GI_50_) of *F. standishii* extracts against human solid tumor cell lines. MW = methanol/water; M = methanol; DM = dichloromethane/methanol; D = dichloromethane; E = ethyl acetate; W = water. Green bars: MW derived fractions; yellow bars: M derived fractions; Orange bards: DM derived fractions.

In order to get an insight on the antiproliferative activity of the extracts, the nine dried samples (Table 1) underwent a primary screening using our [15] of the NCI protocol [16]. The panel of human solid tumor cell lines used for this first screening comprised the non-small cell lung cancer A549 and SW1573, the breast cancer HBL-100 and T-47D, and the cervix cancer HeLa. The maximum test concentration was set at 250 μg/mL and the exposure time 48 h, according to NCI guidelines [16,17]. All samples induced antiproliferative activity against all cell lines, although with diverse potency. Figure 2 depicts the results expressed as 50% growth inhibition (GI_50_) in a more visual manner by using the GI_50_ range plot. Taken as a whole, the GI_50_ values enabled an arrangement of the extracts in the following decreasing order of activity: MW > M > DM. It is notorious that the dichloromethane partition fraction of the polar extract (MW) from *F. standishii* exhibited a significant antiproliferative activity and resulted the most active one (GI_50_ = 8.5–39 μg/mL) among the nine assayed extracts.

Also, for the MW extract, the ethyl acetate partition fraction (MW_E_) induced moderate antiproliferative effects (GI_50_ 25–50 μg/mL), whilst the water partition fraction (MW_W_) was the least active (GI_50_ 39–152 μg/mL). In contrast, for the M extract, the most potent fraction was the ethyl acetate fraction, followed by the water and the dichloromethane. Finally, the three partition fractions of the DM extract did not display significant differences in antiproliferative activity.

Given our interest in the pharmacognostic study of extracts that could provide relevant phenotypic changes in treated cells, the hydroalcoholic fractions (MW) were prioritized for further testing. At this point, several phenotypic experiments could be envisioned to get a hint on the overall mode of action and give information about possible cellular pathways.

### 2.3. Effects on Reproductive Viability

**Figure 3:**
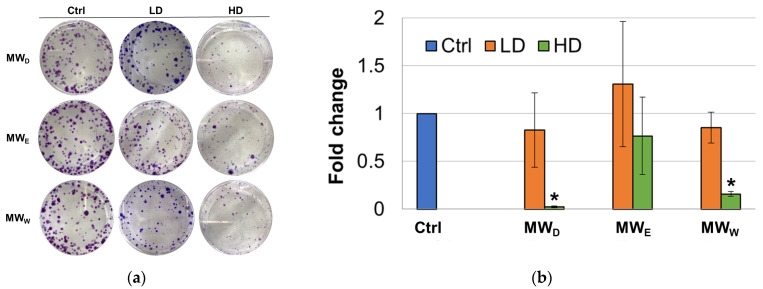
(**a**) Representative images of clonogenic assay of *F. standishii* MW fractions against HeLa cells. (**b**) Normalized average number of colonies after exposure for 7 days to MW fractions at two doses (low dose, LD; high dose, HD). MW_D_ (dichloromethane fraction) = 3 and 8.5 µg/mL; MW_E_ (ethyl acetate fraction) = 8 and 25 µg/mL; MW_W_ (water fraction) = 14 and 43 µg/mL. Error bars: SD after 3–4 replicates. * *p* < 0.05. Ctrl: untreated cells.

Although this assay was initially developed as a tool to determine the effectiveness of radiation on the survival and proliferation of mammalian cancer cells [18], the clonogenic assay is a widely known test to evaluate the ability of compounds to interfere with the survival and growth ability of cells after long-term exposure [19]. To achieve this goal, clonogenic assays were performed by incubating HeLa cells with the three MW extract fractions for 7 days. For this test, two concentrations were considered (high dose, HD, low dose, LD). Considering this extended exposure time, only the GI_50_ (HD) and GI_50_/3 (LD) concentrations. The images taken depict differences in the size of the colonies between the control and the treatment groups (Figure 3). At the LD, the dichloromethane (MW_D_) and the water (MW_W_) fractions were able to produce a decrease in the number of cell colonies formed when compared to untreated cells. The results for the ethyl acetate fraction (MW_E_) were not statistically significant. At the HD, all three fractions were able to produce a decrease in the number of cell colonies formed when compared to untreated cells. Fractions MW_D_ and MW_W_ were the most potent and the difference when compared to control cells resulted statistically significant.

### 2.4. Effects on Cell Migration

Cell migration is an essential process for living organisms because it is involved in embryological development, tissue formation, immune defense or inflammation. However, cell migration is also relevant for cancer progression. Thus, cell migration assays provide insights into the metastatic potential of cell lines and help to discover compounds with potential antimigration capacity in vivo [20]. The in vitro wound healing is an easy and cheap way to preliminarily assess cellular movement. However, this technique has been described in the literature using different conditions.

The non-small-cell lung cancer cell line A549 is considered as a highly metastatic cancer cell line and represents a good in vitro model to assess the antimigratory properties using the wound healing assay [21]. In our group, we observed that cell migration assays require low serum conditions to avoid cell growth influence. With A549 cells, we found that using 1–2.5% serum the closure was maximal at 24 h while cell growth was kept to a minimum [15,22].

**Figure 4:**
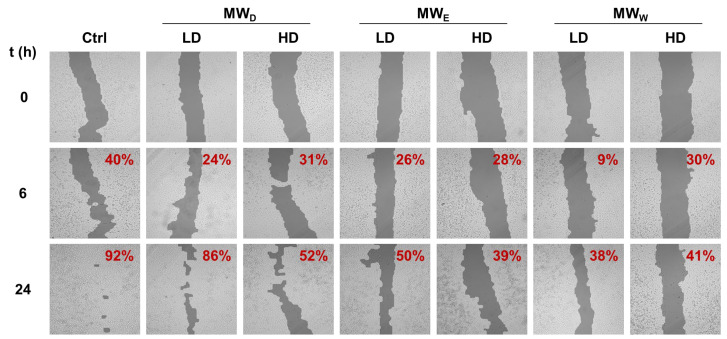
Representative images of wound closure assay of *F. standishii* MW fractions against A549 cells. Images were taken after 6 and 24 h of exposure to MW fractions at two doses (low dose, LD; high dose, HD). MW_D_ (dichloromethane fraction) = 5 and 14 µg/mL; MW_E_ (ethyl acetate fraction) = 17 and 50 µg/mL; MW_W_ (water fraction) = 49 and 146 µg/mL. Wound closure percentage with respect to time = 0 is given on the right top corner of each image. Ctrl: untreated cells.

To study the influence on cell migration, A549 were treated with the three MW fractions for 24 h. Likewise, two concentrations were considered, GI_50_ (LD) and 3 × GI_50_ (HD). The closure of the wound was monitored after 6 and 24 h of exposure. The results are given in Figure 4. Overall, the extracts reduce cell migration as denoted by the reduced closure of the wound when compared to the control. The results did not show relevant differences in potency among the three fractions assayed. Our findings are consistent with a previous study of the ethanolic extracts from *F. magellanica* and *F. triphylla* [10].

### 2.5. Induction of Oxidative Stress

Elevated levels of Reactive Oxygen Species (ROS) lead to cellular oxidative stress. In cancer treatment, many anticancer drugs induce oxidative stress, which is a common mechanism for causing cancer cell death. Previous studies demonstrated that ethanolic and aqueous extracts from *F. magellanica* and *F. triphylla* inhibit intracellular ROS production in 3T3 and HaCaT cells [10]. In that study, ROS formation was experimentally induced by exposing the 3T3 fibroblast and HaCaT keratinocyte cells to peroxyl radicals from 2,2′-azo-bis(2-amidinopropane) dihydrochloride (AAPH). In contrast, the present study explores the potential of *F. standishii* extracts to induce ROS production. In animal cells, mitochondria are the primary source of ROS formation, which is frequently associated with mitochondrial dysfunction and/or damage. Mitochondrial malfunction often precedes cell death via the intrinsic (mitochondrial) apoptotic pathway.

**Figure 5:**
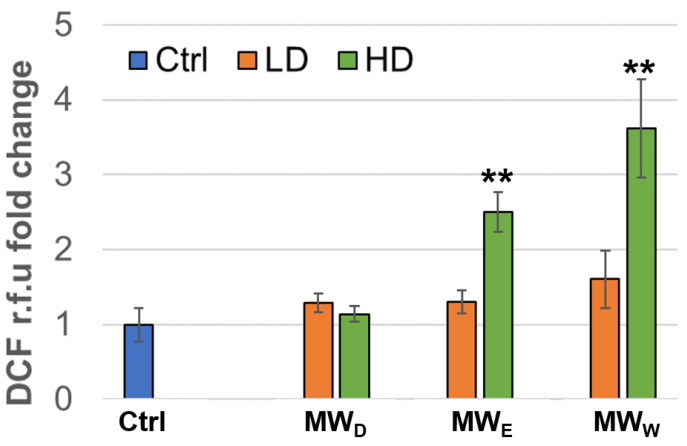
Normalized ROS production expressed as DFC fluorescence intensity relative to that of the control of HeLa cells after exposure for 3 h to MW fractions at two doses (low dose, LD; high dose, HD). MW_D_ (dichloromethane fraction) = 0.023 and 0.043 µg/mL; MW_E_ (ethyl acetate fraction) = 0.075 and 0.125 µg/mL; MW_W_ (water fraction) = 0.129 and 0.215 µg/mL, respectively. Error bars: SD after 3–4 replicates. ** *p* < 0.01. Ctrl: untreated cells.

To determine the induction of intracellular ROS, we used the DCF method (2′,7′-dichlorofluorescin diacetate). HeLa cells were exposed to the plant fractions for 3 h at two doses (a high and a low concentration). As shown in Figure 5, the water (MW_W_) and the ethyl acetate (MW_E_) fractions were able to induce significant ROS production in HeLa cells, as measured by normalized DCF fluorescence intensity compared to untreated cells. This apparent contradiction with the reported scavenging effect [10] can be explained in terms of dosage. The ROS-scavenging capacity of *Fuchsia* species previously reported IC_50_ values around 25 µg/mL, whereas our extracts induced ROS at much lower doses (0.125 µg/mL and 0.215 µg/mL, Figure 5).

### 2.6. Induction of DNA Damage

**Figure 6:**
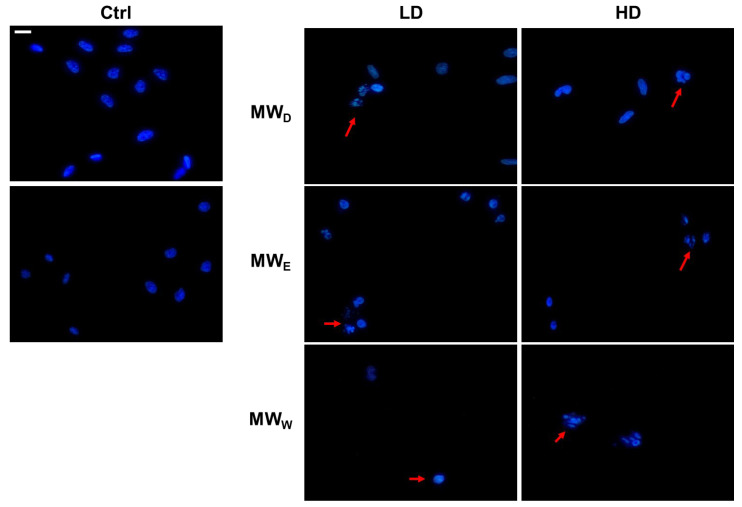
Representative images of HeLa cells stained with DAPI (blue) after exposure for 24 h to MW fractions at two doses (low dose, LD; high dose, HD). MW_D_ (dichloromethane fraction) = 0.023 and 0.043 µg/mL; MW_E_ (ethyl acetate fraction) = 0.075 and 0.125 µg/mL; MW_W_ (water fraction) = 0.129 and 0.215 µg/mL, respectively. Red arrows point to cells with DNA fragmentation. Scale bar = 40 µM. Ctrl: untreated cells.

One of the hallmarks of cell death caused by drugs is nuclear shape modification. Hence, we carried out an evaluation using DAPI staining to obtain more information about the possible nuclear modifications induced by the three fractions derived from the aqueous extract of *F. standishii*. HeLa cells underwent exposure to the extracts at two doses and for a 24 h period. Figure 6 shows the changes induced in nuclei morphology. The nuclei of HeLa cells exposed to MW_D_ fraction displayed a characteristic DNA fragmentation pattern, a feature typical of apoptosis. Apoptotic nuclei appear slightly smaller than normal nuclei. In contrast, for cells exposed to fractions MW_E_ and MW_W_, the observation was nuclear fragmentation typical of cell death by necrosis. In this process, cell death occurs rapidly due to the direct effect of stress on the cell. Necrosis has been described as an uncontrolled event, which agrees with the results observed in the induction of ROS production (Figure 5), where these two fractions induce a significant increase in ROS levels.

**Figure 7:**
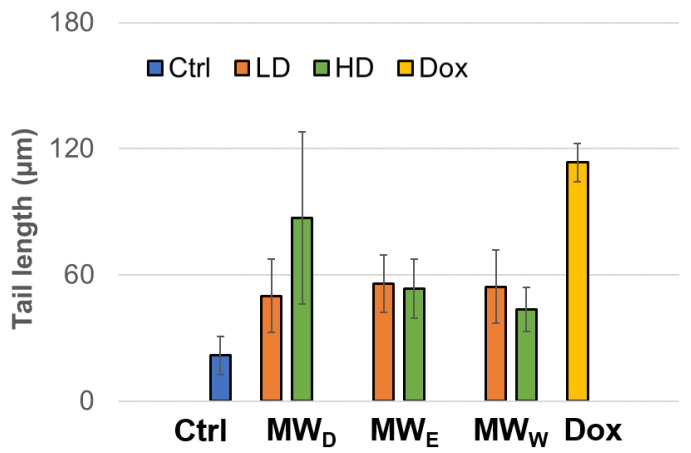
Genotoxicity of HeLa cell line exposed to three fractions of the aqueous extract of *Fuchsia standishii* for 24 h: MW_D_ (dichloromethane fraction) = 2.83 and 4.25 µg/mL; MW_E_ (ethyl acetate fraction) = 8.33 and 12.5 µg/mL; MW_W_ (water fraction) = 14.33 and 21.5 µg/mL, respectively. Each bar represents the mean of three duplicate experiments. Ctrl: untreated cells; LD: low dose; HD: high dose; Dox: doxorubicin.

Another test for assessing DNA damage is the comet assay, also known as single-cell electrophoresis (SCGE), which is a robust and sensitive technique that allows the detection and quantification of various types of DNA damage in individual cells. This assay can identify and evaluate single-strand breaks (SSBs) and double-strand breaks (DSBs), alkali-labile sites, DNA-protein cross-links, as well as oxidative damage in genetic material. The results obtained after exposure of the HeLa cell line to fractions from the aqueous extracts of *F. standishii* are presented in Figure 7. A clear induction in genotoxic damage is observed. These findings closely correlate with those obtained in other complementary assays in this study, particularly those related to the induction of ROS and cell viability. This pattern suggests that the detected DNA damage is directly associated with increased intracellular ROS levels, which in turn could trigger or enhance the cell death pathways observed under these conditions.

### 2.7. Exploring the Mode of Cell Death

**Figure 8:**
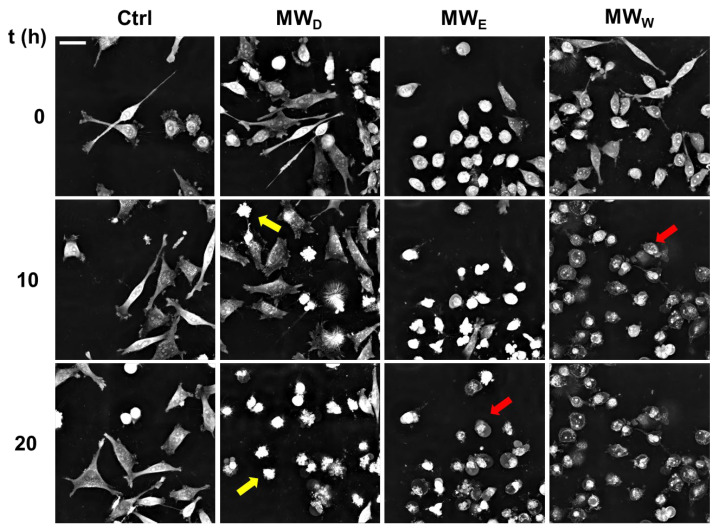
Representative snapshots of HeLa cells exposed for 20 h to MW fractions. MW_D_ (dichloromethane fraction) = 0.043 µg/mL; MW_E_ (ethyl acetate fraction) = 0.125 µg/mL; MW_W_ (water fraction) = 0.215 µg/mL. Yellow arrows indicate the presence of apoptotic cells. Red arrows indicate the presence of necrotic cells. Scale bar = 40 µM. Ctrl: untreated cells.

While DAPI staining helped visualizing nuclear shape modifications, live cell imaging of HeLa cells allowed following the kinetics of cell death. Live cell imaging allows following up the dynamical behavior of cells exposed to compounds that are missing in single time end-point observations. We studied the evolution of HeLa cells exposed to the three fractions for 20 h. The doses were 0.043, 0.125, and 0.215 µg/mL for MW_D_, MW_E_, and MW_W_, respectively. Images were taken every 15 min (Supplementary Videos S1–S4). The analysis of the images confirmed the results obtained with DAPI staining (Figure 6). As shown in Figure 8, the MW_D_ fraction induced cell death by apoptosis, whilst MW_E_ and MW_W_ fractions killed cells by necrosis.

**Figure 9:**
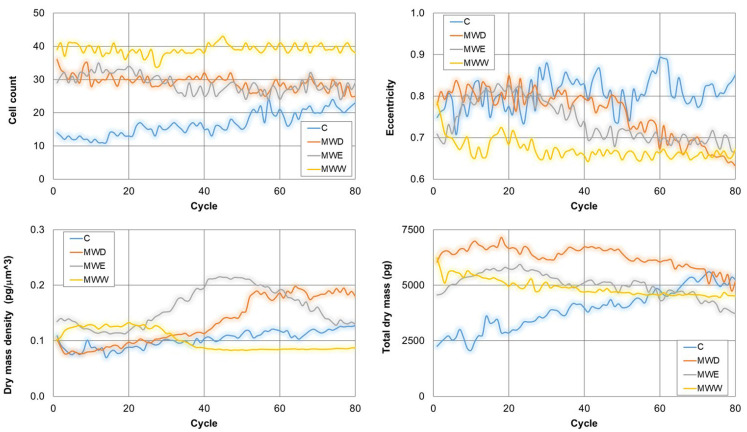
Kinetics of representative phenotypic parameter for untreated HeLa cells, and cells exposed to fractions for 20 h. MW_D_ (dichloromethane fraction) = 0.043 µg/mL; MW_E_ (ethyl acetate fraction) = 0.125 µg/mL; MW_W_ (water fraction) = 0.215 µg/mL. C: untreated cells.

Continuous live-cell imaging helps in the kinetic analysis of phenotypic changes in a population at single cell level. This technique changes the paradigm in the study of cell response to potential bioactive compounds [23]. Figure 9 shows the evolution on time of the cell count, the eccentricity, the dry mass density and the total dry mass. Thus, cell count only increased for control cells. Thus, a clear stop in cell growth occurred in all treatments. In addition, eccentricity decreased in all treated samples. This parameter provides a hint on cell morphology. Values close to 1 indicate spread cells and close to 0 indicates round shape. The results show that the MW_W_ fraction reduced eccentricity from the early stages of the treatment. For the other two fractions, the effects were evident in the middle of the exposure. Dry mass density is a parameter that increases when cells enter apoptosis and decreases when cells dye by necrosis. Consistent with the videos, our results indicate that the MW_W_ fraction induced necrosis, whilst the MW_D_ fraction produced the compartmentalization of cellular organelles in apoptotic bodies and the shrinkage of cells resulting in apoptosis. Interestingly, the MW_E_ fraction induced initially an increase in the dry mass density, which reduced gradually 10 h after exposure. This is consistent with a cell death mechanism of apoptosis followed by necrosis. Finally, the total dry mass decreased for all treatments. This parameter correlates with the rupture of the membrane and the release of the cellular content to the extracellular environment, a signal of transition of final stages of cell death.

### 2.8. Chemical Profile of the Hydroalcoholic Extract and Its Derived Fractions

**Figure 10:**
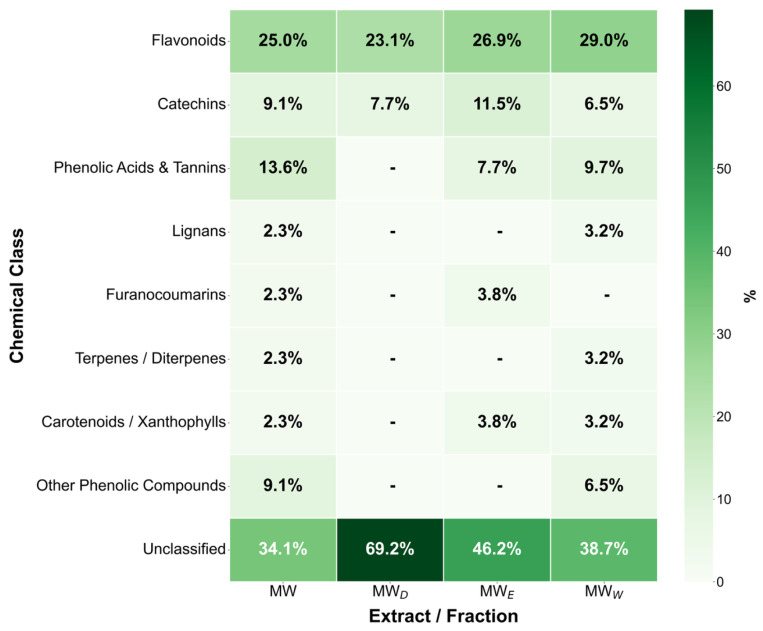
Percentages of Key compound classes derived from UHPLC-DAD-MS^3^ analysis of *Fuchsia standishii* hydroalcoholic extract and derived fractions.

The final step supporting the anticancer evaluation of the *F. standishii* extract was the initial chemical analysis of its hydroalcoholic extract (MW) and derived fractions (MW_D_, MW_E_, and MW_W_). The objective was to determine the qualitative composition of specialized metabolites using UHPLC-DAD-MS^3^ [24]. Notably, this work constitutes the first preliminary chemical study of extracts and fractions from the *F. standishii* species. Compound identification (summarized in Table 2 and Figure 10) was provisionally based on mass spectral analysis (molecular masses and MS/MS fragmentation patterns) compared against available literature and databases. The initial aqueous extract (MW) yielded a total of 47 secondary metabolites, primarily belonging to the flavones, tannins, and chalcones phytochemical classes. Fractionation achieved a significant differential separation: 31 metabolites were detected in MW_W_, 25 in MW_E_, and only 10 in MW_D_. Importantly, the MW_W_, which contained the highest metabolite load, correlated with the highest induction of ROS. Of special interest is the MW_D_ fraction, which, despite having the lowest number of metabolites, exhibited the highest apoptotic activity. Detailed characterization of MW_D_ revealed that most of its identified metabolites belong to the flavonoid (mainly the flavone subtype) and diarylheptanoid classes. Compounds in these classes are widely recognized for their significant antitumor, anti-inflammatory, and antioxidant properties [25,26,27]. Therefore, the selectivity and high concentration of these potent flavonoids and diarylheptanoids in the MW_D_ fraction strongly suggest they are directly responsible for the potent apoptosis induction observed in the bioassays. However, it is important to note that 15 of the 47 metabolites detected could not be identified, underscoring the limitations of previous phytochemical studies in this species.

## 3. Discussion

As a member of the *Onagraceae* family, *F. standishii* is hypothesized to yield new compounds for treating various diseases. This potential is consistent with the cytotoxic and antiproliferative effects reported for its parent species *F. magellanica* [10,11]. Therefore, in this work, we explored the potential of the aerial parts *F. standishii*, excluding the flowers, for cancer treatment.

In this study, the polar extracts from the aerial parts of *F. standishii* yielded the highest quantities (Table 1), directly indicating a predominance of polar compounds. The extraction yield is a critical pharmacognostic parameter, as it provides an initial assessment of the plant’s potential as a valuable source of bioactive compounds and guides the prioritization of species selection [15,29,30]. Intriguingly, the high activity observed in the less-polar MW_D_ fraction (Figure 2) contrasts with the higher yield of the polar fractions. This strongly suggests that the most potent antiproliferative compounds are non-polar to medium-polarity specialized metabolites. Given our goal to identify extracts that elicit relevant phenotypic changes in treated cells, the hydroalcoholic fractions (MW) were prioritized for the subsequent pharmacognostic study. This initial selection enabled the design of various secondaty phenotypic assays to elucidate the overall mode of action and suggest potential cellular pathways, specifically focusing on relevant hallmarks not reported for *F. standishii*.

When we investigated the extract’s effect on cell migration (Figure 4), we observed only a modest reduction in movement. Given that cell migration assays assess a sample’s antimetastatic potential [31], the antimetastatic effects of *F. standishii* are therefore considered low. In stark contrast, the long-term results from the cell viability (clonogenic) assay showed that the compounds are able to induce significant antiproliferative effects (Figure 3). Intriguingly, the MW_D_ fraction was the least active in the migration study yet the most potent in the clonogenic assay. These initial data suggested a differential mode of action among the MW_D_, and the other two fractions (i.e., MW_E_, and MW_W_). This differential pattern was further supported by the ROS assay (Figure 5), the results of which could be correlated to DNA damage observed with DAPI staining (Figure 6). Indeed, DAPI staining revealed two distinct modes of cell death: apoptosis was induced in HeLa cells treated with the MW_D_ fraction, while necrosis occurred after exposure to fractions MW_E_ and MW_W_. While the comet assay (Figure 7) showed no clear difference in activity among the fractions, this result is consistent with DNA damage occurring due to either apoptosis or necrosis. The final evidence confirming this differential mode of action was provided by the live cell imaging study, which conclusively verified the induction of apoptosis by MW_D_ and necrosis by the other two fractions. This difference in the mode of cell death strongly reinforces the hypothesis that each fraction contains a distinct profile of compounds, a finding that was ultimately confirmed by the chemical profiling (Table 2).

The intriguing connection between the traditional uses of *Fuchsia standishii* (antihypertensive, antacid, and relaxant) and its potent observed anticancer activity is likely explained by shared anti-inflammatory and antioxidant pathways. Many of the ailments addressed by traditional remedies—including hypertension and gastrointestinal issues—often have underlying inflammatory and oxidative stress components. The plant’s *Onagraceae* family is known for its anti-inflammatory and cytotoxic properties, which initially led to the hypothesis that the traditionally used extracts may possess antiproliferative properties, given that chronic inflammation is a precursor to many chronic diseases, including cancer. This hypothesis is strongly supported by the chemical profile of the active fraction (MW_D_), which contains specialized metabolites like epigallocatechin gallate and isoquercitrin. These compounds are widely recognized in pharmacology for their potent antioxidant, anti-inflammatory, and cytotoxic effects. Therefore, the traditional uses appear to be rooted in the same anti-inflammatory and oxidative pathway-modulating compounds that are now demonstrating therapeutic potential against cancer. This translational overlap highlights the pharmacological continuity of the plant’s activity, validating its ethnobotanical relevance and suggesting its potential value extends from symptomatic relief to the management of complex chronic diseases.

In this work, we significantly expand the limited current knowledge available for *F. standishii*. Prior literature primarily focused on the flowers, reporting the presence of polar compounds such as flavonols, flavones, hydroxybenzoic acid, hydroxycinnamic acid, and specific metabolites including pelargonidin 3-*O*-β-glucopyranoside, quercetin III glucoside, ellagic acid, rutin, and anthocyanins. Similar constituents were also reported in the flowers of *F. regia* [32].

To the best of our knowledge, our study represents the first detailed investigation of the polar extracts from the aerial parts of *F. standishii*. Crucially, we are the first to demonstrate the presence of substances capable of inducing apoptosis or necrosis in human solid tumor cell lines. This significant finding opens the way for further investigations directed towards the isolation, structural characterization, and biological evaluation of the unidentified compounds to fully explore their potential as anticancer agents.

## 4. Materials and Methods

### 4.1. Plant Material

The selected species was *F. standishii* J.Harrison (syn. *F. hybrida*) belonging to the *Onagraceae* family. Its vernacular name in Ecuador is pena pena and in other parts of the world it is known as queen’s earrings or aljaba. The collection of the plant material was in the province of Loja, in Santiago sector with the location according to GPS coordinates 3°47′22″ South–79°17′20″ West, during the end of January. Dr. Fanny Tinitana (Universidad Técnica Particular de Loja) identified the specimen. A voucher specimen of the species exists in the herbarium of the Universidad Técnica Particular de Loja under code HUTPL 14444. To collect the specimen, we obtained the research permit from the Ministerio del Ambiente, Agua, y Transisición Ecológica del Ecuador with code MAAE-ARSFC-2022-2001.

For this study, we collected the aerial parts of the plant with the exception of the flowers. The collected pant material underwent drying in an airy oven at 40 °C (Agriculture Dryer–model DY-330H) for one week. The dried plant material remained stored at room temperature in the darkness until further use.

### 4.2. Extraction of Plant Material and Fractionation

The preparation of the extracts from *F. standishii* followed the methodological approach described previously [15]. Reagent-grade solvents (Merck, Darmstadt, Germany) and Milli-Q (ultrapure water) were used. Each maceration step of the plant material was carried out for 24 h, using constant stirring and employing magnetic stirrers to optimize extraction. Additionally, the entire process was performed in complete darkness to prevent the degradation of light-sensitive compounds. Thus, the grinded plant material from *F. standishii* (100 g) underwent maceration in 600 mL of methanol–water (1:1, *v*/*v*, MW). After filtration, a second maceration of the plant residue in 600 mL of methanol took place, followed by filtration and final maceration in 600 mL of dichloromethane-methanol (1:1, *v*/*v*, DM). All maceration processes proceeded in the darkness and at room temperature. The obtained three mother liquors (L_MW_, L_M_, and L_DM_) underwent concentration under reduced pressure to evaporate the less volatile solvent. The concentrates of L_M_ and L_DM_ received water (300 mL) to resuspend the concentrate. In a separating funnel, the mother liquors (L_MW_, L_M_, and L_DM_) undertook sequential liquid–liquid extraction with dichloromethane (300 mL) and ethyl acetate (300 mL), respectively. Overall, the process afforded three liquid phases (dichloromethane, ethyl acetate and water) from each mother liquor. The liquid phases were concentrated under reduced pressure to evaporate the less volatile solvent, resuspended in water and lyophilized to afford the corresponding dry sample. Thereafter, each sample was weighed and stored at 4 °C until further processing.

### 4.3. Cells, Culture and Plating

Prof. Godefridus J. Peters (VU Medical Center, Amsterdam, The Netherlands) kindly provided the human solid tumor cells lines used in this study: A549 (lung), HBL-100 (breast), HeLa (cervix), SW1573 (non-small-cell lung), and T-47D (breast). Cells were maintained in 35 mm dishes. The cell culture medium was RPMI 1640 supplemented with 5% FBS and 2 mM L-glutamine. Incubation of cells was at 37 °C, 5% CO_2_, and 95% humidified air. For fluorescent and live cell imaging assays the conditioned medium used RPMI 1640 without phenol red. For all tests, exponentially growing cells were trypsinized and resuspended in antibiotic-containing medium (100 units of penicillin G and 0.1 mg of streptomycin per mL). Cell suspensions were counted and dilutions were made to obtain appropriate cell densities for inoculation into the appropriate material: dishes or (microtiter) plates.

### 4.4. Antiproliferative Tests

The antiproliferative tests in 96-well plates followed the NCI guidelines [16] using the SRB assay [33] with the following cell seeding densities: 2500 cells/well for A549, HBL-100, HeLa, and SW1573, and 5000 cells/well for T-47D. Stock sample was treated with DMSO at an initial concentration of 100 mg/mL. Test samples were prepared by decimal serial dilutions of the stock sample to give a final test concentration of 250, 25 and 2.5 µg/mL. The incubation time was 48 h. The optical density of each well was measured at 530 (primary) and 620 nm (secondary) using a microplate reader (Power Wave XS, BioTek, Winooski, VT, USA). The antiproliferative activity, expressed as 50% growth inhibition (GI_50_) values, was calculated according to the NCI formulas [16]. The criteria used to categorize the cytotoxicity of the extracts on the NCI’s protocol was as follows: GI_50_ ≤ 20  µg/mL = highly cytotoxic, GI_50_ ranged 21–200 µg/mL = moderately cytotoxic, and GI_50_ > 201  µg/mL = weakly cytotoxic.

### 4.5. Clonogenic Assay

The clonogenic assays in 6-well plates required a cell seeding density of 450–800 HeLa cells/well [19]. On the next day after plating, the medium was renewed, and samples were added at the indicated dose. The exposure time was seven days after which time the medium was removed, and the colonies were fixed with ethanol for subsequent staining with crystal violet (0.5% *w*/*v*, deionized water). Colonies were counted using AutoCellSeg (1.0.7), a MATLAB (2016b) implementation for automatic segmentation [34].

### 4.6. Cell Migration Assay

The non-small-cell lung cancer cell line A549 is a highly metastatic cancer cell line that represents a good in vitro model to assess the antimigratory properties using the wound healing assay [35]. Cell migration studies in 24-well plates followed the protocol of the wound healing (scratch) assay [36] with a density of 50,000 cells/well. When cells reached >90% confluence, a mark was drawn on the outside bottom of each well, to find easily the reference point when taking pictures. For each well, a scratch across the cell monolayer was made perpendicularly to the mark using a sterile 200 µL tip. Then, the cell culture medium was replaced with new medium containing 2.5% FBS. This allows us to diminish the interference caused by cell proliferation during the time of assay, evaluating only cell migration. Next, the samples were added at the indicated doses. Pictures were taken with a brightfield microscope (Axiovert 40 CFL, Zeiss, Oberkochen, Germany) at one magnification (5×) using the software ZEN 2012 (Blue edition v1.1.0.0) at diverse time intervals (0, 6 and 24 h since the scratch formation). For the quantification of cell migration, TScratch (1.0), an image software based in MATLAB (2016b), was used adapting threshold of images to measure the area of the wound made [37].

### 4.7. Determination of Reactive Oxygen Species

ROS detection in 96-well dark-walled transparent bottom plates followed the widely used DCF fluorescent assay [38]. For these assays, HeLa was the cell line of choice. Cell seeding density was 5000 cells/well. On the next day after plating, the cell culture medium was replaced with RPMI medium without phenol red and the cell permeable probe 2′,7′-dichlorodihydrofluorescein diacetate (DCFH-DA, 40 μM) was added. Then, samples were added at the indicated doses, and the exposure time was 3 h. After exposure, the fluorescence intensity was measured at excitation/emission wavelengths of 492/520 nm (Varioskan LUX multimode plate reader, Thermo Fischer Scientific, Waltham, MA, USA).

### 4.8. DNA Damage Assays

#### 4.8.1. DAPI Staining for Nuclear Morphology and Cell Damage Assessment

HeLa cells were seeded on top of sterile coverslips placed in a 6-well plate (100,000 cells/well). After overnight incubation, the samples were added at the indicated concentration. After exposure, cells were fixed using a paraformaldehyde solution (1 mL, 4% in PBS) for 10 min at room temperature. To reduce the PFA background, cells were washed for 15 min with a NH_4_Cl solution (50 mM in PBS) and then rinsed with PBS. Next, a Triton solution (0.1% in PBS) was used for 10 min to permeabilize the cells. Then, the cells were washed three consecutive times with PBS for 5 min each. DAPI (750 μL, 1 μg/mL in methanol) was added for 10 min under light shaking. The DAPI solution was recovered, and the cells were rinsed again 2 to 3 times maintaining dark conditions. The coverslips were then mounted on a microscopy slide over a drop of Mowiol mounting medium and sealed with nail polish to prevent evaporation. At least 6 random fields were imaged using the 20× and 40× magnification in an epi-fluorescence microscope (Axiolab 5, Zeiss, Germany). Finally, quantification of the mean fluorescence of DAPI-stained cells was performed using FIJI/ImageJ (1.54f) Software (NIH, USA).

#### 4.8.2. Comet Assay

The comet assay began with the preparation of microscope slides, which were coated with a base layer of 150 µL of 1% (*w*/*v*) normal melting point agarose (NMPA) (Invitrogen). The treated cells (with a low dose of GI_50_/3 and a high dose of GI_50_/2 of each fraction) were resuspended in 1% (*w*/*v*) low melting point agarose (LMPA) (Sigma (St. Louis, MO, USA)). Specifically, 20 µL of the cell suspension was mixed with 150 µL of 1% LMPA, and 75 µL of this mixture was spread onto the slides. After initial solidification, a sealing layer of 130 µL of 1% (*w*/*v*) LMPA was added, and the samples were refrigerated for 10 min at each step to ensure immobilization. Once immobilized, the samples were subjected to lysis in an alkaline solution (Day Lysis) for a period of 1 h to 15 days to denature the membranes and release the DNA. This solution was prepared with 90 mL of Mother Lysis, 9 mL of DMSO (9% *v*/*v*), and 1 mL of Triton X-100 (1% *v*/*v*). The stock solution was formulated as 1.25 M NaCl, 63.3 mM EDTA, and 4.95 mM Tris, adjusted to pH 10. Subsequently, the plates were incubated in an electrophoresis chamber containing Alkaline Unwinding and Electrophoresis Buffer for 20 min (unwinding). This buffer consisted of 0.3 M NaOH and 2.5 mM EDTA, adjusted to pH > 13. Electrophoresis was performed immediately afterward under these conditions with strict control at 25 V and 300 mA for 20 min. This step allowed the damaged DNA to migrate out of the nucleus, forming comet-like structures. Finally, the samples were neutralized with a Neutralization Buffer composed of 80 mM Tris (pH 7.5), dehydrated with methanol, and stained with ethidium bromide for visualization. Quantification of genotoxic damage was performed by measuring the ‘tail length’ of the comets using the specialized software Comet Assay IV (4.2) and a fluorescence microscope, analyzing a minimum of 200 comets per plate in duplicate samples [39].

### 4.9. Continuous Live-Cell Imaging

A CX-A imaging platform microscope (Nanolive SA, Lausanne, Switzerland) was used to study the kinetics of cells exposed to the samples. HeLa cells were seeded at a density of 50,000 cells/well onto a 35 mm cell culture imaging dish (ibidi GmbH, Gräfelfing, Germany) and treated at the indicated doses before the acquisition of the images (acquired every 15 min for 20 h). Data obtained was transferred to FIJI (NIH, USA) for image analysis. STEVE (2.2.1.2162) software (Nanolive SA, Tolochenaz, Switzerland) was used for the analysis of the refractive indices and obtention of the phenotypic parameters.

### 4.10. Phytochemical Analysis (UHPLC-DAD-MS^3^ Analysis)

Ultra High Performance Liquid Chromatography-Diode Array Detector-Tandem Mass Spectrometry (UHPLC-DAD-MS^3^) analysis of the aqueous extract and its fractions (MW_D_, MW_E_, and MW_W_) was carried out by a previously reported method [28], with slight modifications. Briefly, 10 mg of each sample was dissolved in 1 mL of methanol-water (1:1, *v*/*v*) and subjected to UHPLC using a Kinetex XB-C18 column (150 mm × 2.1 mm × 1.7 μm, Phenomenex, Torrance, CA, USA). Elution was carried out at a flow rate of 0.3 mL/min using a solvent system composed of 0.1% formic acid in water (A) and 0.1% formic acid in acetonitrile (B), with the following gradient: 0 min−3% B, 60 min−26% B, and 120 min−95% B. Compounds were detected by UV-Vis spectra recording in the range of 190 to 600 nm, and chromatograms were monitored at 280 nm. Identification and characterization were based on mass spectra obtained in positive and negative ion modes, in combination with UV-Vis absorption maxima, and were complemented by consulting databases such as PubChem and PhytoPub for the detection and identification of compounds previously reported in the species *Fuchsia standishii.*

## 5. Conclusions

The current study provides the first comprehensive investigation into the antiproliferative potential of extracts derived from the aerial parts of *F. standishii*, a species of high ethnobotanical importance in southern Ecuador. Our research successfully demonstrated that *F. standishii* possesses a potent pharmacological profile that plausibly extends beyond its documented traditional uses (e.g., antacid, relaxant, and treatment for high blood pressure). The strongest anti-proliferative activity was concentrated in the MW_D_ fraction, exhibiting high cytotoxicity against a panel of solid tumor cells. Mechanistically, this activity was confirmed to occur via the induction of apoptosis and genotoxic damage linked to increased oxidative stress in the cells.

In summary, the MW_D_ fraction is a plausible source of novel anti-cancer agents due to its ability to induce apoptosis in tumor cells. These results validate the continued ethnobotanical investigation of *F. standishii* and strongly support the next crucial step: bioguided isolation and structural elucidation of the specific compounds responsible for the potent antiproliferative activity.

## Figures and Tables

**Table 1:** Yield (%) of *Fuchsia standishii* extracts and their fractionations.

Mother Liquor	Fraction			Total Data
	D	E	W	
MW	0.2	1.8	26.8	28.8
M	0.2	1.3	4.1	5.6
DM	0.04	0.3	0.3	0.64

MW = methanol:water; M = methanol; DM = dichloromethane:methanol; D = dichloromethane; E = ethyl acetate; W = water.

**Table 2:** UHPLC-DAD-MS^3^ analysis of *Fuchsia standishii* hydroalcoholic extract and derived fractions.

Peak	RT (min)	MS [M+H]^+^	MS [M−H]^−^	MW	MW_D_	MW_E_	MW_W_	Putative Compound	Data Base
1	2.2	407.08		✔				Piceatannol 4′-glucoside	PubChem
2	2.3		481.18	✔			✔	3′-(6″-Galloylglucosyl)-phloroacetophenone	PubChem
3	2.9		331.21	✔				1-O-Galloyl-beta-D-glucose	PubChem
4	3.7		169.04	✔				Gallic Acid	PubChem
5	4.3		633.17	✔		✔	✔	Corilagin	PubChem
6	4.8		565.12	✔			✔	Isoginkgetin	PubChem
7	8.3	187.95		✔				Angelicin	PhytoHub and PubChem
8	8.4		315.33	✔			✔	Royleanone	PubChem
9	10.5		487.29	✔			✔	6″-O-Acetylglycitin	PhytoHub and PubChem
10	11.4		451.45	✔			✔	Aspalathin	PubChem
11	13.7		182.97	✔	✔	✔	✔	U.C. ^a^	–
12	15.6		783.18	✔			✔	U.C. ^a^	–
13	16.3		297.32	✔		✔	✔	U.C. ^a^	–
14	16.3	162.93		✔				Indole-3-carboxylic acid	PubChem
15	17.5		577.23	✔		✔	✔	Kaempferitrin	PubChem
16	18.3	291.07		✔				(+)-Catechin	PhytoHub and PubChem
17	18.4		289.31	✔		✔		(+)-Epicatechin	PubChem
18	19.1		417.43	✔			✔	(+)-Syringaresinol	PhytoHub and PubChem
19	22.7		431.44	✔			✔	Genistin	PubChem
20	25.9		785.12	✔		✔	✔	U.C. ^a^	–
21	27.4		513.26	✔			✔	U.C. ^a^	–
22	32.4		493.34	✔	✔	✔	✔	U.C. ^a^	–
23	33.3		479.2	✔		✔	✔	U.C. ^a^	–
24	34.1		305.24	✔		✔	✔	Gallocatechin	PhytoHub and PubChem
25	36		615.17	✔		✔	✔	6″-O-Galloylquercimeritrin	PubChem
26	37.7		609.21	✔		✔	✔	Hesperidin	PhytoHub and PubChem
27	38.2		463.16	✔	✔	✔	✔	2′-Hydroxyisoorientin	PubChem
28	39.3		463.19	✔	✔		✔	Isoquercitrin	PubChem
29	40.5		457.38	✔	✔	✔	✔	Epigallocatechin gallate	PubChem
30	40.5	435.2		✔				5-Hydroxyaloin A	PubChem
31	41.1		477.23	✔		✔	✔	Hesperetin 7-O-glucuronide	PubChem
32	41.9	435.11		✔				Quercetin 3-arabinoside	PubChem
33	43.5		599.21	✔		✔		Kaempferol galloylhexoside	[28]
34	44	449.15		✔	✔	✔	✔	Luteolin 7-O-glucosid	PhytoHub and PubChem
35	45.4		419.13	✔		✔	✔	Kaempferol 3-O-arabinoside	PubChem
36	47.4		599.22	✔		✔	✔	Neoxanthin	PhytoHub
37	50.2	213.05		✔				U.C. ^a^	–
38	52.1		711.54	✔		✔	✔	U.C. ^a^	–
39	52.3	227.02		✔				U.C. ^a^	–
40	52.6		505.31	✔		✔	✔	U.C. ^a^	–
41	53.5		541.39	✔		✔		U.C. ^a^	–
42	67.9		545.24	✔	✔	✔		U.C. ^a^	–
43	73	219.08		✔				U.C. ^a^	–
44	80.3		487.56	✔	✔	✔	✔	U.C. ^a^	–
45	89		649.5	✔	✔	✔	✔	U.C. ^a^	–
46	92.9		297.41	✔		✔	✔	U.C. ^a^	–
47	98		455.47	✔	✔			U.C. ^a^	–

^a^ UC = unidentified compound. ✔: compound detected.

## Data Availability

The original contributions presented in the study are included in the article/Supplementary Material, further inquiries can be directed to the corresponding author.

## Supplementary Materials

The following supporting information can be downloaded at https://www.mdpi.com/article/10.3390/plants14243779/s1, Figure S1. UHPLC-DAD chromatograms for MW, MW_D_, MW_E_, and MW_W_ fractions, Video S1: Untreated HeLa cells (control), Video S2: HeLa cells exposed to MW_D_, Video S3: HeLa cells exposed to MW_E_, Video S4: HeLa cells exposed to MW_W_.

## Abbreviations

The following abbreviations are used in this manuscript:

DAPI	4′,6-Diamidino-2-phenylindole
DCF	Dichlorofluorescein
DCFH-DA	Dichlorodihydrofluorescein diacetate
HD	High dose
LD	Low dose
NCI	National Cancer Institute
ROS	Reactive oxygen species
RPMI	Roswell Park Memorial Institute
UHPLC-DAD-MS^3^	Ultra-High-Performance Liquid Chromatography, Diode Array Detector, Mass Spectrometry with a third stage of fragmentation
