# Supplementary material for: Antiproliferative Effects of Polar Extracts of the Aerial Parts of Fuchsia standishii J. Harrison
Source: Plants (Basel). 2025 Dec 11;14(24):3779. doi: 10.3390/plants14243779 (PMC12736804; doi:10.3390/plants14243779)
Supplement: Supplementary file 1 [file plants-14-03779-s001.zip › Figure S1. Plants_Fuchsia_SI.pdf]

Antiproliferative effects of polar extracts of the aerial parts of *Fuchsia standishii* J.Harrison

María I. Ramírez [1,2], Aday González-Bakker [1], Adam N. Khan [1], Adrián Puerta [1] and José M. Padrón [1,*]

[1] *BioLab, Instituto Universitario de Bio-Orgánica Antonio González (IUBO-AG), Universidad de La Laguna, PO Box 456, E-38200 La Laguna, Spain*

[2] *Departamento de Ciencias de la Salud, Universidad Técnica Particular de Loja. San Cayetano alto, ZIP 11-01-608 Loja, Ecuador*

SUPPLEMENTARY INFORMATION

Figure S1. UHPLC-DAD chromatograms.

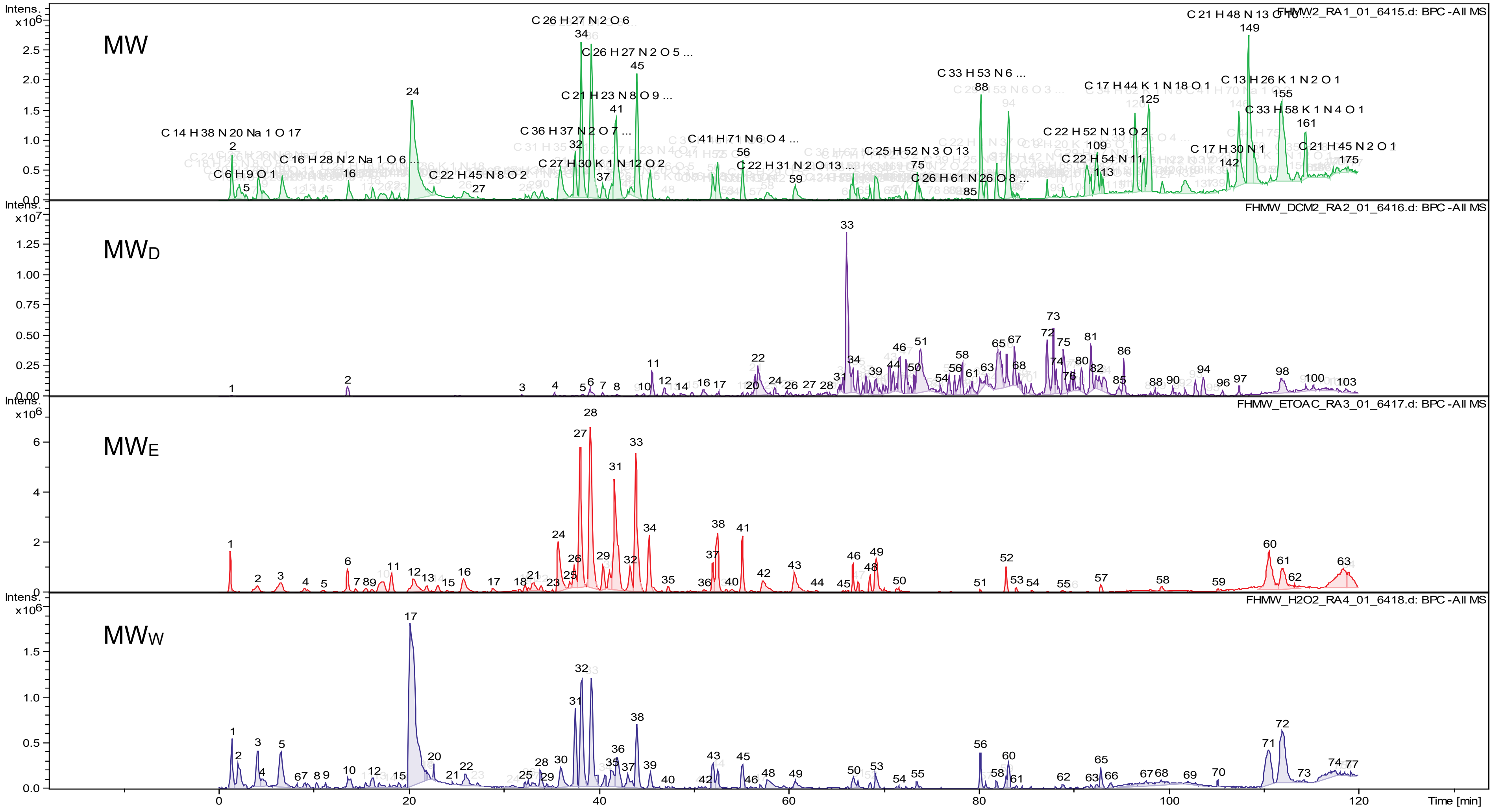

Figure S1. UHPLC-DAD chromatograms for MW, MW_D, MW_E, and MW_W fractions.
